# Implementing comprehensive prevention of mother-to-child transmission and HIV prevention for South African couples: study protocol for a randomized controlled trial

**DOI:** 10.1186/1745-6215-15-417

**Published:** 2014-10-27

**Authors:** Deborah Jones, Karl Peltzer, Stephen M Weiss, Sibusiso Sifunda, Ntabozuko Dwane, Shandir Ramlagan, Ryan Cook, Gladys Matseke, Vincent Maduna, Andrew Spence

**Affiliations:** Department of Psychiatry and Behavioral Sciences, University of Miami Miller School of Medicine, Executive Offices, 1120 NW 14th ST Suite 1463, Miami, Florida 33136 USA; HIV/AIDS, STIs and TB (HAST) Research Programme, Human Sciences Research Council (HSRC), Private Bag X41, Pretoria, 0001 South Africa; Department of Psychology, University of the Free State, Room 202, Campus North Avenue, Bloemfontein, 9300 South Africa; ASEAN Institute for Health Development, Mahidol University, 25/25 Phutthamonthon Road 4, Salaya, Phutthamonton, Nakhon Pathom 73170 Thailand

**Keywords:** HIV, PMTCT, South Africa, Couples, Male involvement

## Abstract

**Background:**

In rural South Africa, only two-thirds of HIV-positive pregnant women seeking antenatal care at community health centers took full advantage of ‘prevention of mother-to-child transmission’ (PMTCT) services in 2010. Studies generally support male involvement to promote PMTCT, but the nature and impact of that involvement is unclear and untested. Additionally, stigma, disclosure and intimate partner violence pose significant barriers to PMTCT uptake and retention in care, suggesting that male involvement may be ‘necessary, but not sufficient’ to reduce infant HIV incidence. This study expands on a successful United States President's Emergency Plan for AIDS Relief (PEPFAR)-supported PMTCT couples intervention pilot study conducted in the Mpumalanga province, targeting HIV-positive pregnant women and their partners, the primary objective being to determine whether male partner involvement plus a behavioral intervention will significantly reduce infant HIV incidence.

**Methods/design:**

The study follows a cluster randomized controlled design enrolling two cohorts of HIV-positive pregnant women recruited from 12 randomly assigned Community Health Centers (CHC) (six experimental, six control). The two cohorts will consist of women attending without their male partners (n = 720) and women attending with their male partners (n = 720 couples), in order to determine whether the influence of male participation itself, or combined with a behavioral PMTCT intervention, can significantly reduce infant HIV infection ante-, peri- and postnatally.

**Discussion:**

It is our intention to significantly increase PMTCT participation from current levels (69%) in the Mpumalanga province to between 90 and 95% through engaging women and couples in a controlled, six session ante- and postnatal risk-reducing and PMTCT promotion intervention addressing barriers to PMTCT (such as stigma, disclosure, intimate partner violence, communication, infant feeding practices and safer conception) that prevent women and men from utilizing treatment opportunities available to them and their infants. Based upon the encouraging preliminary results from our pilot study, successful CHC adoption of the program could have major public health policy implications for containing the epidemic among the most vulnerable populations in rural South Africa: HIV-positive pregnant women and their infants.

**Trial registration:**

ClinicalTrials.gov NCT02085356 (registration date: 10 March 2014).

**Electronic supplementary material:**

The online version of this article (doi:10.1186/1745-6215-15-417) contains supplementary material, which is available to authorized users.

## Background

Prevention of mother-to-child transmission of HIV (PMTCT) strategies have dramatically reduced infant morbidity and mortality associated with HIV, as well as significantly improving maternal health [1]. Computer modeling estimates indicate that 90 to 95% participation in PMTCT strategies plus effective pharmacologic regimens would reduce infant HIV incidence to the World Health Organization’s (WHO) goal of under 5% worldwide [2, 3]. Guidelines for PMTCT have been implemented in most HIV-affected countries [4], however, PMTCT program failure and dropout occurs at all stages of the ante-, peri- and postnatal process [5]. Not all pregnant women are tested for HIV [6], not all women receive treatment [7] and not all mothers provided with medication take it themselves or give it to their newborns [8]. Safer infant feeding guidelines are not always followed, not all infants born to HIV-infected mothers are tested and treated [3] and mothers and newborns often miss postpartum clinic visits [9]. Globally, it is estimated that two thirds of newborn deaths could be avoided by utilization of existing maternal, newborn and child health policy packages such as family planning, PMTCT, skilled birth attendance or clean childbirth, early identification of illness, safer infant feeding and HIV treatment through antiretroviral therapy (ART) [10].

In South Africa, although progress has been recorded in the implementation of PMTCT programs, these improvements have, for the most part, occurred in urbanized areas, with rural areas remaining at unacceptably high levels of MTCT [11]. The lack of paternal support, stigma, lack of testing or disclosure, gender iniquities and inequalities, intimate partner violence (IPV), lack of PMTCT information, clinic access and poor retention in care have been identified as major challenges to PMTCT effectiveness in South Africa’s rural areas [12–15]. The Mpumulanga province, the location of this trial, is a predominantly rural area with one of the lowest rates of PMTCT participation and one of the highest antenatal clinic (ANC) HIV prevalence rates (37%) in the country.

Increasing male participation in antenatal care has been proposed in order to enhance PMTCT uptake [16], however, while men are traditionally the sexual and reproductive decision-makers in South Africa, the impact of male involvement in PMTCT remains unclear and untested [17]. Men possess general HIV knowledge but lack specific information regarding PMTCT [18] and may feel unable to attend ANCs due to work schedules [19]. Men also may regard ANC health facilities as being ‘generally unfriendly’ to them [20]. Men are perceived as decision-makers in the home, and feel their position is undermined if they are expected to attend a ‘women’s clinic program’, leading them to decline to attend ANC visits with their partners [21]. A review of studies in Africa involving men in antenatal care concluded that male support, as well as involvement, may be required to increase PMTCT uptake [22].

Additionally, as many women in sub-Saharan Africa spend a considerable time of their adult lives pregnant [23], one of the major components of the PMTCT continuum is the provision of reproductive health choices to enable either the prevention of unintended pregnancies, or appropriate planning for intended future pregnancies [24]. Factors limiting the use of family planning are similar to those preventing PMTCT uptake, such as fear of HIV disclosure, stigma and IPV [25], including fears of judgmental healthcare providers with negative attitudes towards childbearing by HIV-seropositive women [26]. When women do discuss fertility plans with providers, the extent to which safer conception methods are included is unclear [27]. In Cape Town, whilst over 30% of HIV-positive women wanted additional children, and 60% in Johannesburg planned to conceive in the next year, in both regions, most had never had a conversation with a healthcare worker on these issues [28].

The ‘Protect Your Family’ trial tests the effectiveness of a piloted behavioral intervention to significantly increase adherence to the PMTCT protocol among rural HIV-positive pregnant women, simultaneously determining whether the intervention conducted both with or without the participation of male partners will have an additive or synergistic (or negative) impact on PMTCT uptake. A full factorial clinic-randomized design is utilized, in which Phase one enrolls women (all of whom have male partners) alone to receive the intervention or time-matched attention control condition, and Phase two enrolls women together with their male partners, who receive a comparable men’s intervention (or control sessions). It is hypothesized that women receiving the intervention will be significantly more likely to adhere to PMTCT protocol elements and, consequently, significantly less likely to have an infant test HIV seropositive as compared to those women in the control condition. Additionally, it is hypothesized that the inclusion of male partners during the second phase of the study will further increase adherence to the PMTCT protocol among the experimental condition mothers.

### Pilot study

A one-year pilot project enrolling 239 couples provided the foundation for this trial. Pregnant women and their partners from ANCs in the Mpumalanga province of South Africa were followed from month four of pregnancy to three months postpartum. Project aims were to increase male involvement in ANC to promote PMTCT and to reduce unprotected sex during pregnancy. Men participated in both experimental and control conditions. The experimental condition included a gender-concordant group intervention based on sexual risk reduction and PMTCT promotion, and the attention-control condition included time-matched usual antenatal care. Although the number of women with HIV (n =76) was too small to derive meaningful statistical comparisons on medication uptake and transmission of HIV to infants, the clinical outcomes were sufficiently compelling to provide guidance and direction for the development of this trial. Medication uptake was assessed via dried blood spot (DBS); HIV medication was detected in 75% and 92% of experimental condition mothers and infants, respectively, as compared to 50% and 75% of those in the control condition [29]. Four infants were HIV-positive by polymerase chain reaction (PCR) at six weeks (one experimental, three control). Additionally, experimental participants were found to have decreased their amount of unprotected sex, increased their HIV-related knowledge and had reduced incidences of IPV at follow-up in comparison with control participants [30]. Qualitative data from both genders reflected concerns about serostatus disclosure and communication.

## Methods/designs

### Study design

**Figure 1:**
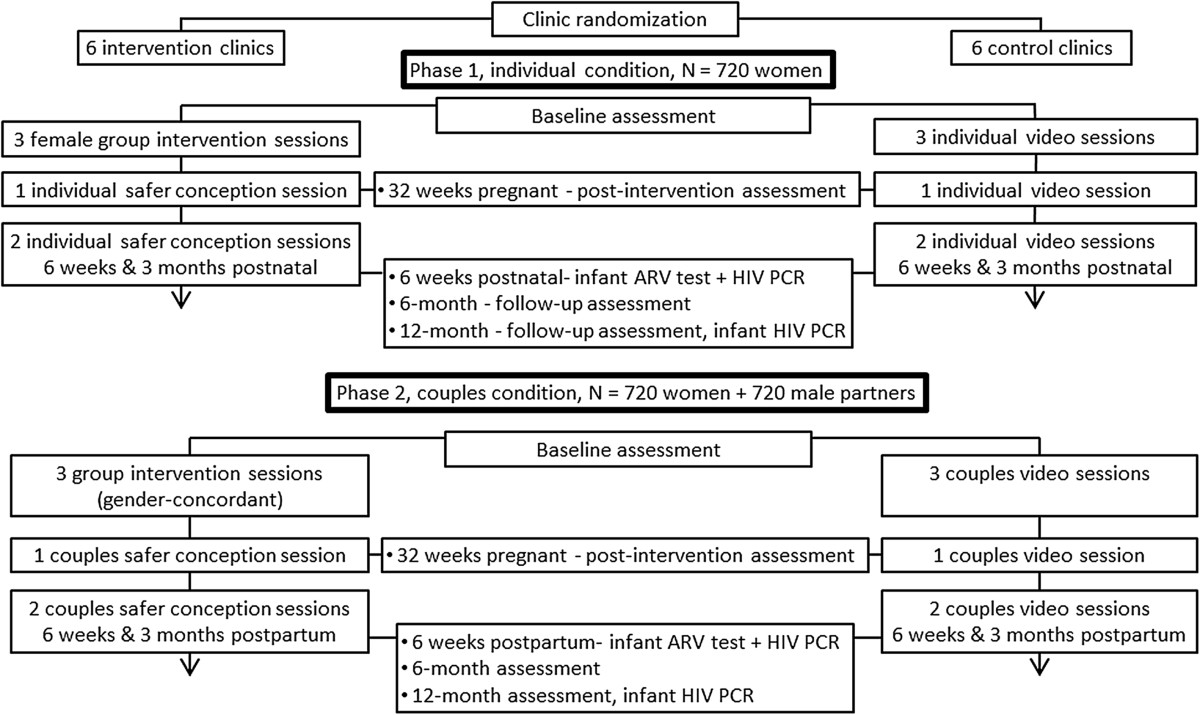
**Study flow diagram for participants in the study.** Antiretroviral; N = Population size.

This study is a clinic-randomized controlled trial using a 2 × 2 × 5 comparison: Phase (women only or couples) × condition (experimental or control) × time (assessments given at baseline, 32 weeks pregnant, and six weeks, six months and 12 months postpartum). In addition to assessments, participants attend three group and one individual or couples counseling intervention (or control) sessions prior to birth, and two individual or couples counseling sessions postpartum. Figure 1 presents a flow diagram for participants in this study.

### Site selection

Prior to initiation of study recruitment, all clinics from the Gert Sibande and Nkangala Districts in the Mpumalanga province were reviewed in consultation with the Provincial Department of Health. Eligible CHCs clinics met South African criteria for PMTCT sites, including on-site daily HIV counselling and testing (HCT), ART distribution and cluster of differentiation 4 (CD4) testing, ante and postnatal counseling on infant feeding, infant HIV testing, two or more trained PMTCT staff and two counselors and a support group for HIV-positive mothers and pregnant women. Twelve CHCs were randomly assigned (see below) as intervention sites or standard of care sites, stratified by antenatal care clinic case load in the upper 50th percentile of MTCT rates (>13%). Selected CHCs were engaged through meetings with study staff and District and Provincial Health Officials. All 12 selected CHCs agreed to participate, and were visited by study personnel to review the objectives of the study with CHC staff members and discuss implementation strategies within the existing CHC framework.

### Randomization

The twelve CHCs were matched in a 1:1 ratio according to patient census and average ANC volume, and one clinic in each pair was randomly assigned to the experimental or control condition using a computer program written by the data manager. The matched clinics were then assigned to the opposite condition. The randomization process was carried out by four people. The first conducted the computer-generated randomization assignments stratified by clinic size (selected a seed for the random number generator, ran the program, and completed the table of condition assignments). The second implemented the assignments, providing a table of all clinic site assignments to study personnel. The third activated each intervention site individually, and the fourth activated each control site individually.

### Blinding

Only the Human Sciences Research Council (HSRC) study staff activating and overseeing the sites were aware of site assignment. All assessments will be conducted using an audio computer-assisted self-interview (ACASI) program. As such, participants enter their data themselves and are blind to their assignment. Following randomization, clinic sites were activated individually, and clinic staff are blinded to the condition. Training for clinic study staff was conducted by condition, and clinic study staff conducting the study are also blind to clinic randomization status. Finally, data analysis to evaluate study outcomes will be blinded to the clinic’s status as an intervention or control intervention arm.

### Training of study staff members

Study staff at the CHC sites underwent formal training on the study protocol, informed consent, protection of human subjects, recruitment, assessment and use of ACASI technology, with an in-depth review of the meaning of each item in the assessment instruments presented by ACASI, presented by the University of Miami (UM) and HSRC investigators. Experimental condition staff attended a five-day training course that included an intensive review of the ‘Protect Your Family’ intervention manual, the PMTCT protocol and use of cognitive behavioral (CB) intervention strategies in the intervention, as well as how to manage sensitive issues (such as serostatus disclosure, IPV, gender dynamics, sexual risk reduction and safer conception practices). Following the training, all staff received additional supervision at their CHC sites on the study protocol for data collection. In addition, experimental condition staff will receive additional guided training and practice on the intervention under the supervision of the intervention coordinator who will act as leader and then co-leader of the intervention at each experimental CHC site for the first two cohorts. Thus, each experimental clinic staff person is currently conducting two sequences of group sessions and individual counseling sessions under the supervision of the HSRC coordinator.

Control condition staff received an identical one-day training session on the use of ACASI technology and a four-hour orientation to the protocol to enable them to conduct time-equivalent group sessions comprised of childhood disease prevention and adult health hazard videotapes (for example measles, diarrhea management and immunizations).

### Participant recruitment and enrollment

**Figure 2:**
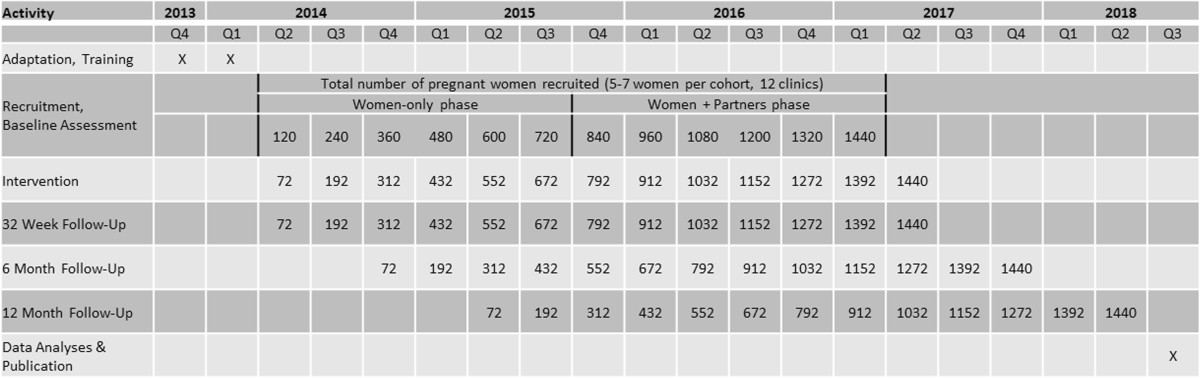
**Study time line and assessment points.**

The total number of study participants (n = 2,160) will be 720 women in phase one and 720 couples (n = 1440) in phase 2; each CHC will recruit 60 women, followed by 60 couples, over 30 months. The study began recruitment in April 2014, and all participants are expected to be enrolled by May 2017. Figure 2 presents a timeline of anticipated study activities, including recruitment and follow-up.

Per South African PMTCT protocol, all women receive pre- and post-HCT and, if HIV positive, referral for CD4 assessment and ART. Potential candidates are referred to the study assessor by PMTCT/HCT staff after completing initiation of ART. Those who are interested in participation are screened for eligibility, provide written informed consent, and are enrolled in the study. Participants are compensated between 50 and 100 South African Rand (approximately US$5 to 10) per assessment for their time and transportation, and are provided snacks during group and individual counseling sessions.

Eligible women are HIV-seropositive pregnant women with partners, between 8 and 24 weeks pregnant (typical time of entry into ANC care) and aged 18 years or older. During phase one, these women are enrolled alone. In phase two, both women and their partners will be enrolled. Male partners in the couples phase will be invited to attend by their female partners following initial contact with the recruiter. Those agreeing to participate will be enrolled following provision of informed consent, maintaining confidentiality of the serostatus of both couple members. Per the standard of care, male partners will be encouraged, but not required, to undergo HCT. For the purposes of this study, primary male partners are defined as husband, current baby’s father, current male sexual partner or trusted male friend actively involved in the mother’s life. All couples will be screened individually and both members will each be asked a series of three rotating questions which will identify them as a couple.

Persons who are actively psychotic (auditory or visual hallucinations) or intoxicated (for example under the influence of alcohol of illegal drugs) are not eligible for the study and will be referred for treatment. Following resolution of symptoms, these persons may become eligible for the study. There are no exclusions based on literacy as all assessments will be administered using ACASI.

The study conforms to the Helsinki Declaration concerning human rights and informed consent. Ethical approval was obtained from the University of Miami, Human Subjects Research Office, HSRO study number 20130238, and the Human Sciences Research Council Research Ethics Committee protocol number REC 4/21/08/13.

### Intervention condition

Intervention participants receive the PMTCT standard of care plus three prenatal weekly two-hour gender-specific (male or female, between five and seven participants) group sessions followed by one individual counseling session and two monthly individual (women only) or couples counseling sessions (one prenatal, two postpartum) led by study-trained clinic staff (see Figure 1, study flowchart). The ‘Protect Your Family’ intervention is a manual, closed, structured behavioral risk-reduction program targeting prevention of vertical transmission, the importance of adherence to PMTCT and medication use, HIV testing of family members and prevention of transmission of HIV, stigma, serostatus disclosure, partner communication, IPV, safe infant feeding, safer conception, family planning and dual method sexual barrier use.

### Group sessions

During group sessions, participants receive CB skill training addressing the key components of each session, for example, how cognitions relate to anticipated outcomes and thereby predict behavioral change. Group members are encouraged to problem solve, providing supportive feedback and peer mentorship on session topics. Participants’ role play communication strategies and complete partner communication and negotiation homework using strategies practiced in the sessions. Participants receive a week’s supply (approximately seven) of condoms following each session. During phase two, session topics for men’s and women’s groups are comparable, but place different emphasis on gender relevant topics, for example, women focus on PMTCT protocol and medication adherence and men focus on HIV testing, alcohol and drug use (men’s sessions do not address topics from the perspective of having a partner with HIV, and do not jeopardize their partner’s HIV status).

Group session one focuses on reviewing information regarding PMTCT, protection from HIV transmission, partner involvement and becoming familiar with communication strategies for sensitive topics including HIV serostatus disclosure, mutual testing, alcohol use, stigma and avoiding IPV. The session concludes with a relaxation session. Men address the above topics with a special focus on undergoing HCT. CB skill training heightens participant awareness of their reactions to PMTCT and communication.

Group session two again addresses positive communication and utilizes role playing, and continues with discussion of HIV status disclosure, prevention of IPV, and CB skills for coping with anger and arguing. The session then addresses PMTCT, ART and male involvement during pregnancy. Men focus on HIV testing, alcohol and drug use and communication. CB skills are used to address anxiety regarding HIV status disclosure.

Group session three addresses testing for HIV to enhance treatment and prevent transmission. The discussion continues on healthcare visits as an element of PMTCT, infant feeding and planning for delivery. Family planning, safer conception and the use of dual methods of contraception following birth are discussed and a role play is conducted. Men focus on conflict resolution and anger management, communication and reducing and/or avoiding IPV. Participants are encouraged to share experiences with disclosure and problem-solving, and are guided in applying cognitive restructuring skills in discussing safer conception negotiation, including family planning post-partum.

### Counseling sessions

Three structured one-hour sessions are led by study-trained CHC staff and delivered to individuals or couples in a one-on-one format. The initial antenatal session is followed by two additional sessions that will occur at six weeks and three months postpartum (during phase two, the two postnatal sessions will include male partners). Information concerns adherence to the PMTCT protocol, such as infant feeding practices and medication and reproductive decision-making, including fertility planning and safer conception practices. Each session enhances motivation to adhere to the PMTCT protocol (the necessity to adhere to the protocol throughout the infant’s first six months of life, or longer, as determined by the child’s health and feeding status). Each session also addresses reducing risk behavior related to unintended pregnancy, acquisition of sexually transmitted infections (STI) and prevention of STI and HIV transmission to partners and use of dual methods of protection (consistent use of condoms along with another contraceptive method).

Counseling session one is held at 32 weeks gestation, and provided to women only, regardless of study phase. The first session is an individual session, and reviews PMTCT, transmission and prevention of transmission during and following pregnancy and infant feeding. This session focuses on a personalized review of the PMTCT protocol (adherence to medication for both mothers and infants, infant feeding practices and postpartum infant care visits) and emphasizes the importance of health facility delivery and provision of HIV medication to both mothers and infants at delivery. The session uses motivational interviewing (MI) to address making a plan for infant feeding, family planning and use of antiretroviral (ARV) medication. The session then addresses delivery, and applies MI to guide mothers to make a plan for delivery.

Counseling sessions two and three occur at six and 12 weeks postnatally and are provided to women (phase one) or couples (phase two). Session two content includes a discussion of safer conception practices and family planning using dual barrier methods (counseling sessions two and three address these topics in a manner that will not compromise participants’ confidentiality). The session includes a review of safe infant feeding (for example the importance of breastfeeding for infant health and avoiding mixed feeding), the importance of infant health clinic visits and a discussion of immunizations. MI is used to enhance immunization uptake, use of family planning and safe infant feeding practices. Session three reviews safer sex and family planning and maintaining medication use and appointments for mother and infant, while staying on track with feeding. ‘Well baby’ visits are used as reminders for the infant’s appointments. MI is used to enhance safer sex and family planning.

### Control condition

Control condition participants receive the PMTCT standard of care plus a time-equivalent, group-administered video presentation on health promotion and disease prevention (such as measles, diarrheal management, dysentery and dehydration and immunizations and vaccinations) in three group sessions, followed by one individual and two couple or individual women’s sessions on disease prevention and health promotion.

### Evaluations, assessments and data management

Whenever possible, clinic and biological data will be gathered from antenatal client records and the South African ‘Road to Health’ booklet [31], which is filled out as part of the standard of care for all expectant and new mothers, and contains comprehensive health information for their infants. All female participants are asked to provide their Road to Health booklet at each assessment. Postpartum clinic visits, maternal ART prescription, intrapartum ARV provision, infant HIV status, infant ART provision, infant feeding and infant immunizations are confirmed from the booklet. In the event that the participant does not provide the booklet, this data will be collected from clinic records.

All psychosocial assessments are presented using ACASI technology to enhance disclosure of sensitive information and reduce the impact of low literacy. All study materials were translated into local languages (Zulu and Sotho) prior to study onset. Assessments include both psychosocial assessments and biological and clinic data at study entry, pre-delivery (32 weeks pregnant), six weeks post-delivery, and at six and 12 months postnatal follow-up.

### Primary outcomes

Primary outcomes include infant HIV serostatus and ART adherence for mothers and infants. Infant HIV serostatus is assessed via PCR at six weeks postnatal as part of the South African PMTCT standard of care, and the results will be collected from the Road to Health booklet or clinic records. A second HIV test will be administered at 12 months as part of study participation. ART adherence for mothers will be assessed by blood sampling (DBS) pre-delivery (32 weeks gestation), and infant adherence will be assessed at six weeks postnatal by DBS. DBS will assess the presence or absence of ARV medications for mothers on lifelong and prophylaxis regimens (tenofovir, lamivudine, efavirenz) and infants taking short-term PMTCT ARVs (nevirapine).

### Secondary outcomes

Secondary outcomes include ante- and postnatal clinic attendance, infant feeding, HIV serostatus disclosure, family planning knowledge, attitudes and practices, HIV and PMTCT knowledge, IPV and communication and male HIV testing and engagement in PMTCT. Clinic attendance is collected from the antenatal card and Road to Health booklet as well as a study appointment log, which is provided to all participants to help keep track of study and clinic appointments. Facilitators and clinic staff write their initials next to appointments as they are completed. Infant feeding is assessed using an adaptation of the WHO feeding scale measuring breastfeeding and replacement feeding practices used in the seven days preceding assessment [32]. HIV disclosure is assessed using an adaptation of the Disclosure Scale assessing disclosure among sexual partners, friends and family members as well as factors associated with disclosure [33]. Family planning is assessed using a survey on knowledge, attitudes and use of contraception and safer conception practices. Knowledge items assess perception of risk of transmission to the partner during pregnancy as well as knowledge of the fertility cycle and ideal time to conceive. A conjoint analysis survey is used to assess family planning attitudes. Contraception practices items assess whether the pregnancy was planned or unplanned, whether a provider was consulted prior to pregnancy, current use of family planning and intentions to engage in family planning in the future. HIV and PMTCT knowledge is assessed using an adaptation of the AIDS-Related Knowledge Test; items reflect information about HIV transmission, reinfection with resistant virus, condom use and PMTCT-specific knowledge [34]. IPV and communication are assessed using the Conflict Tactics Scale, assessing conflict resolution style over the current month and previous 12 months [35]. Finally, male HIV testing is collected from self-report, and male engagement is assessed using an adapted form of the Male Involvement Index [36].

### Covariates

In addition to demographic covariates (such as age and education level), information regarding HIV stigma and postpartum depression is collected. Stigma is assessed using an adaptation of the Women Involved in Life Learning from Other Women (WiLLOW) HIV/AIDS Stigma Instrument, measuring perceived and enacted stigma in the home, community, workplace and healthcare settings, and the AIDS-Related Stigma Scale [37]. Postpartum depression is assessed using the Edinburgh Postnatal Depression Scale; pregnant women and new mothers indicate the frequency of depressive symptoms over the past seven days [38].

### Quality assurance and control

Intervention fidelity is continually assessed using audio recordings of intervention sessions and interventionist checklists that are reviewed by the intervention coordinator. A subset of intervention session audio recordings will be reviewed for fidelity by study staff. Over the course of the study, a randomly selected sample of 10% of the total number of sessions will be transcribed by study staff using headphones in private rooms at the HSRC offices and reviewed. Data quality assurance procedures, including reviewing for errors and consistency checks, are completed monthly by the data manager. The quality of biological data will be monitored by the site laboratory under the accreditation standards of the South African National Accreditation Systems.

### Statistical analyses

Preliminary analyses will include descriptive statistics (such as means, standard deviations, frequencies and percentages), as well as t-tests, chi-square tests, and Pearson’s correlations of factors associated with outcomes. Potential confounders are those observed to have moderate associations with outcome variables in preliminary tests of association, using a conservative α of 0.20 for significance testing. These variables will be controlled in analyses as appropriate. Prior to analyses, appropriate variable transformations will be applied to outcome variables in order to satisfy distributional assumptions. SAS (SAS Institute, Inc., Cary, North Carolina, United States) and SPSS (Statistical Packages for Social Sciences, IBM Company, Armonk, NY, USA) will be used for all analyses using a level of 0.05 to determine statistical significance. All study analyses will be on an intention-to-treat basis.

A series of generalized linear mixed models will be conducted in order to test the impact of the intervention on study outcomes, including condition status (intervention versus control), time, and the interaction of time and condition status as the fixed predictors of interest. Random effects will be used to account for the multilevel data structure of repeated observations within persons, persons nested within cohorts and cohorts nested within clinics. Secondary analyses will test the moderating effect of phase (women only versus couples) as well as explore other potential moderators such as HIV-serostatus disclosure, self-efficacy for disclosure, and male partner serostatus. Significant moderators will be retained in the final model. Planned comparisons will be made between conditions or phases at each time and between times within each condition or phase.

In order to test the relative contribution of ART adherence, safe infant feeding and retention in care on infant HIV test outcomes at 12 months, additional analyses will be conducted using generalized linear mixed models in which infant HIV serostatus at 12 months will be the outcome, and ART adherence, safe infant feeding and retention in care will be the fixed predictors of interest. Random effects will be similar to those described above.

### Sample size determination

The sample size was determined by a power analysis for the primary outcome of infant serostatus at six weeks. Averaged clinic data collected during and following the pilot study indicated that approximately 13% of infants will be seropositive at six weeks of age. Using an HIV PCR rate of 13% in the control arm, a power analysis indicated that six sites per condition (six experimental, six control) with an unadjusted sample size of 564 infants per phase would provide 80% power to detect a significant difference between conditions assuming a reduction to 4% in the intervention condition and intracluster correlation coefficients of up to 0.02 (depending on the two rates) with a two-tailed test at the 0.05 level [39]. The sample size of 720 pregnant women per phase was based on our experience in the pilot study, from which we anticipate a 16% miscarriage and infant death rate and a 5% attrition rate over 12 months (n = 156 lost; n = 564 retained).

## Discussion

Despite the availability of an effective PMTCT treatment protocol and infant feeding guidelines designed for PMTCT, uptake in rural South Africa remains suboptimal [40]. The ‘Protect your Family’ trial seeks to enhance uptake pre- and postpartum, with the goal of significantly reducing current vertical transmission rates of 13% or greater to the WHO goal of less than 5%. While there have been behavioral interventions to promote the PMTCT process, as well as studies attempting to illustrate the contribution of male participation to PMTCT uptake, this trial will be the first to determine the relative effectiveness of both strategies, individually or collectively, in promoting PMTCT uptake in rural South Africa. Implementation of the intervention by CHC personnel provides the foundation for rapid translation and scale-up of the program. If successful, the ‘Protect Your Family’ intervention will provide a generalizable, integrated, sustainable model for clinics with high rates of HIV and a high incidence of MTCT to optimize PMTCT program delivery and effectiveness. Scale-up of the program would have major health policy implications for containing the epidemic in two of the most vulnerable affected populations in rural South Africa - HIV-seropositive pregnant women and their infants.

### Trial status

The ‘Protect your Family’ trial is underway and started recruiting participants in April 2014. Recruitment and follow-up are expected to be completed by August 2018.

## Authors’ original submitted files for images

Below are the links to the authors’ original submitted files for images. Authors’ original file for figure 1 Authors’ original file for figure 2

## Abbreviations

ACASI: Audio computer-assisted self-interview
ANC: Antenatal clinic
ARV: Antiretroviral
ART: Antiretroviral therapy
CB: Cognitive behavioral
CHC: Community health centre
DBS: Dried blood spot
HCT: HIV counseling and testing
HIV: Human immunodeficiency virus
HSRC: Human Sciences Research Council
IPV: Intimate partner violence
MI: Motivational interviewing
PEPFAR: The United States President’s Emergency Plan for AIDS Relief
PCR: Polymerase chain reaction
PMTCT: Prevention of mother-to-child transmission
STI: Sexually transmitted infection
WiLLOW: Women Involved in Life Learning from Other Women.
