# Supplementary figures and images for: Implementing comprehensive prevention of mother-to-child transmission and HIV prevention for South African couples: study protocol for a randomized controlled trial
Source: Trials. 2014 Oct 27;15:417. doi: 10.1186/1745-6215-15-417 (PMC4219009; doi:10.1186/1745-6215-15-417)

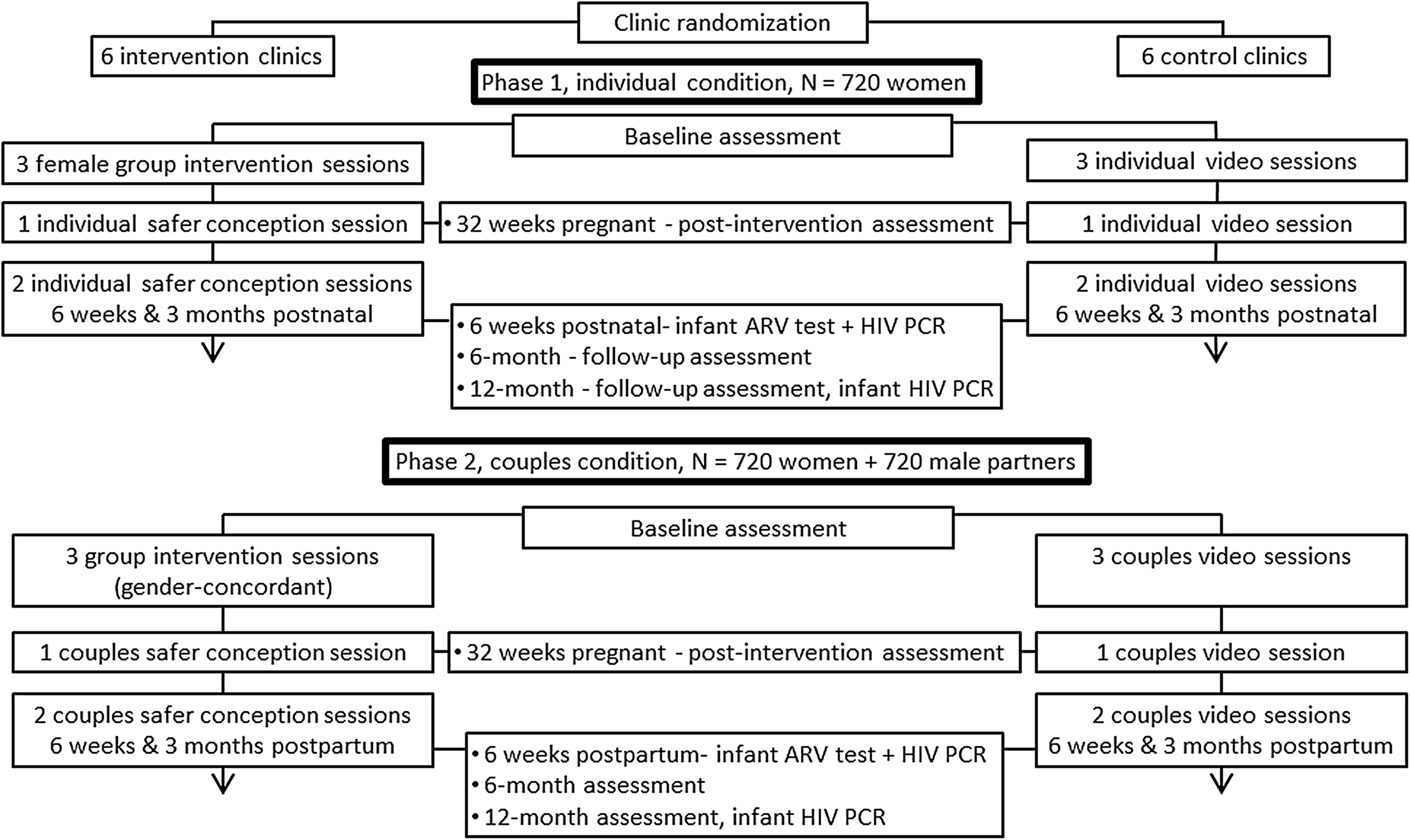

Supplement: Supplementary file 1 — Authors’ original file for figure 1 [file 13063_2014_2273_MOESM1_ESM.tif]

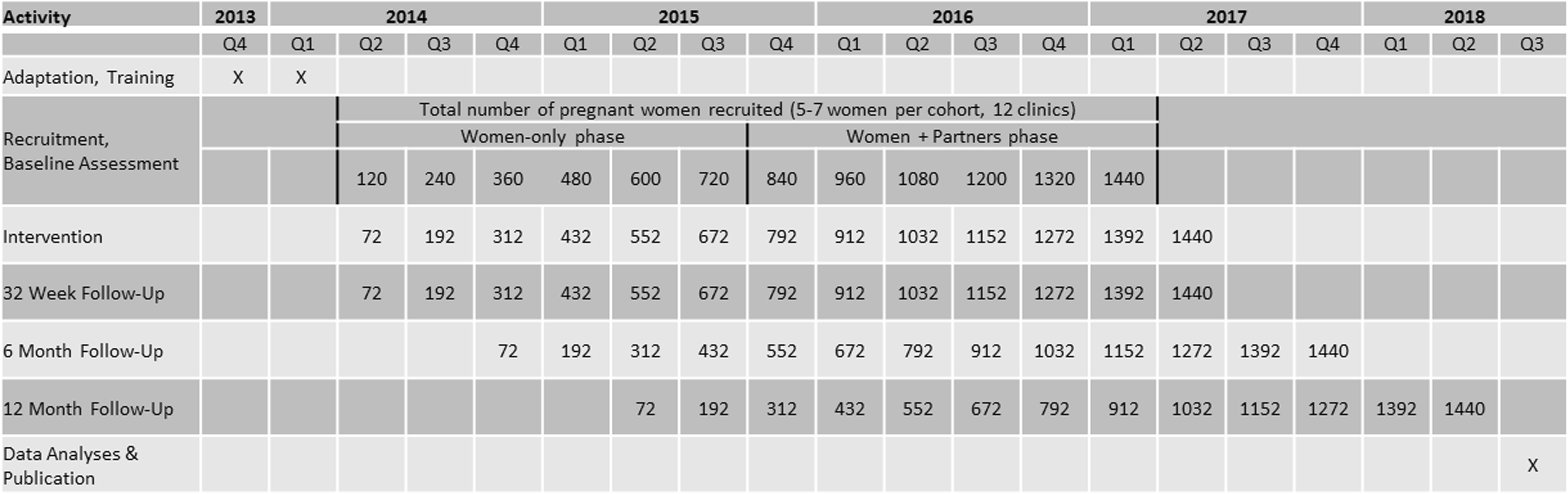

Supplement: Supplementary file 2 — Authors’ original file for figure 2 [file 13063_2014_2273_MOESM2_ESM.tif]
